# Factors affecting young doctors’ choice of medical specialty—A qualitative study

**DOI:** 10.1371/journal.pone.0297927

**Published:** 2024-02-01

**Authors:** Beniamin Michalik, Mateusz Kulbat, Alicja Domagała

**Affiliations:** 1 The J. Dietl Specialist Hospital in Krakow, Krakow, Poland; 2 5th Military Clinical Hospital in Krakow, Krakow, Poland; 3 Institute of Public Health, Faculty of Health Sciences, Jagiellonian University Medical College, Krakow, Poland; University of KwaZulu-Natal College of Health Sciences, SOUTH AFRICA

## Abstract

**Background:**

Young doctors’ choice of medical specialty is essential to maintaining a sufficient health workforce and developing a sustainable healthcare system. The choice of specialization is the result of numerous factors, including doctors’ preferences, population needs, and the number of available residency slots. The aim of this article is to explore the factors taken into consideration by young Polish physicians in choosing their future specialty.

**Methods:**

We have conducted 30 structured interviews with randomly selected recent medical school graduates (17 female and 13 male). The study was conducted from December 2022 until February 2023 using online platforms. Data from in-depth interviews were coded using NVivo Release 1.6.1. Then thematic analysis was performed.

**Results:**

Respondents indicated remuneration and career-associated factors as the main determinants, emphasizing that work-life balance, personal motivations, and the flexibility to choose the type of workplace (hospital vs. outpatient clinic) were even more important. Young doctors expect flexibility in working hours and better working conditions in future work, and these factors are important when choosing a medical specialty. Significant difficulties during the selection of medical specialty include limited residency slots in preferred specializations and lack of knowledge concerning the everyday aspects of work in a particular specialty.

**Conclusions:**

Factors and limitations influencing the choice of medical specialty should be taken into account by policymakers to address the shortages of doctors in deficit specialties. Knowledge about doctors’ preferences regarding their medical specialty could support the development of targeted policies to increase the attractiveness of deficit specialties and reduce the uneven distribution of medical staff.

## Introduction

One challenge facing healthcare systems is sufficiently addressing the health needs of society by properly allocating a limited amount of healthcare professionals in terms of location and medical specialty. In Poland, both the number and the age composition of practicing physicians gives rise to concerns about future supply [1]. The medical fields most affected by a shortage of doctors are internal medicine, family medicine, and psychiatry [2].

In 2012, the Ministry of Health introduced regulations indicating priority medical specialties with serious staff shortages, which are updated annually after consultation with the National Medical Chamber (membership to which is obligatory for all practicing doctors) and other stakeholders [3]. National Medical Chamber participates in the establishment of educational standards, monitors continuous education (CME), and maintains registers of licensed and active physicians. Based on the aforementioned regulations, more residency training opportunities and higher wages have been offered to doctors pursuing these priority “deficit specialties”. Currently, there are 23 priority specialties, including internal medicine, paediatrics, family medicine, geriatrics, and psychiatry [3]. In recent years, the number of specialists in most fields has increased, however, there are still many deficit fields and there are concerns about these shortages increasing in the future [4, 5]. This problem is not only due to the small number of residency slots but also because some specialties are less popular among young doctors, and only some available training slots are filled by residents [6, 7].

Undergraduate education for medical doctors lasts six years, following which all graduates complete a 13-month postgraduate internship and pass the State Medical Examination. Those who pass the exam and finish their postgraduate internship have the full right to practice [4]. Doctors who wish to obtain a specialty must complete postgraduate/specialty training, which lasts between 4–6 years (depending on the specialty) and pass the State Specialization Exam. Recruitment for postgraduate training is conducted twice a year in two full rounds. A ranking list is established based on the results of the qualification process, which considers primarily the outcomes of the State Medical Exam (max. 200 points), as well as other factors like scientific achievements (max. 5 points) and possession of graduate degrees (max. 5 points). In each round of the selection process, applicants are allocated residency slots based on their qualification ranking, their preferences, and the availability of training slots. Each applicant is given the option to either accept or reject the assigned slot. If a slot is rejected in the first round, applicants will have the opportunity to participate in the second round of selection. Since 2023, the Ministry of Health may organize an additional third round for priority specialties [8]

The total number of residency slots (funded by the state budget) is constantly increasing since 2016 but the specializations with the most available slots are not necessarily attractive for young doctors and there is a significant mismatch between the number of residency slots established by the Ministry of Health and the expectations of young doctors [7]. During the autumn 2022 procedure, the Minister of Health awarded 4,218 residency slots; including 2,596 slots in priority specialties. Most residency slots were granted in the fields of family medicine, internal medicine, paediatrics, psychiatry, obstetrics and gynaecology, anaesthesiology and intensive care, general surgery, orthopaedics, neurology, and clinical oncology [9]. Physicians’ career planning is recognized as a complex process, influenced by numerous factors. Introducing “priority specialties,” with better pay for residents did not solve the problem and experts have noted that doctors choose specialty based on the possibility of earning extra money in private practice and the current market situation [10]. However, both Polish [11, 12] and international research [13–19] point out that the issue is much more complicated as there are many factors affecting medical specialty choice. Some of the most often indicated determinants besides economic factors are personal characteristics such as gender [13–16], personal preferences and interests [12–19], job characteristics and working conditions [11–16, 19], and the training process [11–19]. While there are similarities between countries, there is significant variance based on country-specific circumstances [20].

The reasons why some specialties fail to attract a sufficient number of doctors are not fully understood, necessitating further research to comprehensively address this question [7]. The results of our study and new evidence on the young generation’s specialty preferences could provide valuable insights and contribute to developing health policies that aim to increase the attractiveness of priority specialties and improve the uneven distribution of future professionals in countries. Therefore, our research aims to explore the factors taken into consideration by young Polish physicians when choosing their future specialty.

## Methods

### Ethical approval

The study framework was approved by the Bioethical Committee of Jagiellonian University (approval number: 1072.6120.215.2022). Written informed consent was achieved from all participants.

### Study design

The study consists of two phases, the first of which (the qualitative) is presented in this article. In the second step, a nationwide quantitative survey is planned. As there is evidence that decisions regarding the choice of medical specialty often change during medical studies [21], we decided to focus on a group of recent medical school graduates who have either just finished their postgraduate internship or are currently undergoing it.

In the qualitative part, we conducted 30 structured interviews with young doctors who had recently graduated from medical schools in different Polish regions. The interview structure allowed respondents to express their perceptions in their own terms regarding the discussed issue [22]. Our inclusion criteria for interviews were to: 1) be currently undergoing a postgraduate internship or 2) have completed the internship no earlier than 31 October 2022. To ensure adequate reporting, we used the COREQ checklist (Consolidated criteria for reporting qualitative research) (S1 Appendix) [23].

To maximize the credibility of the research, we used random sampling of willing people who fulfilled inclusion criteria. In the first step, in November 2022 we used four nationwide social media groups for young doctors to disseminate information about our research and seek willing participants. 49 people who fulfilled our inclusion criteria volunteered. Next, from this group, we randomly selected 30 people as potential respondents. They were invited for interviews by email or phone. No one refused to participate. This approach fulfilled the seven ways of correct, targeted sampling for qualitative research defined by Palinkas et al. [24]. The interviews were conducted using online platforms (Zoom or MS Teams, depending on participant preferences), and they took place from December 2022 to February 2023. The interviews were carried out in Polish by two authors of this article (BM and MK). Both interviewers are young medical doctors with broad knowledge and interest in the topic and were previously trained in conducting in-depth interviews. At the beginning of each interview, the researchers introduced themselves and clarified the study goals. The interviews were carried out following an interview guide (S2 Appendix). Our participants were guaranteed that their personal data and names would remain confidential when publishing results. On average, the interviews took 30 minutes (the shortest interview lasted 20 min. and the longest lasted 42 min.).

### Study population

Thirty respondents participated in the study (17 female and 13 male), all of whom were between 24 and 28 years old. To be included in our research participants had to be medical school graduates who were either currently undergoing a postgraduate internship (17 respondents) or have finished it no earlier than 31 October 2022 (13 respondents). The characteristics of the participants are presented in Table 1.

**Table 1:** Participant characteristics.

Postgraduate internship	Gender	
	Female (F)	Male (M)
Finished	9	4
Currently undergoing (intern)	8	9
Total number of participants	17	13

#### Data collection and analysis

An interview guide was developed by the research group and piloted before starting research. Depending on the answer to the first question *(Have you already chosen a specialization*?*)* each participant answered two or five open-ended questions. Afterward, all respondents were asked two more open-ended questions. During interviews, detailed field notes were taken and then sent to respondents for their revision. Interview materials were translated from Polish to English by the first author (BM). Detailed field notes were later analysed by all researchers using six phases of thematic analysis [25]. After familiarization with the data, initial codes were created using NVivo Release 1.6.1. Each question was coded separately, first using the automatic coding feature of the NVivo program and then, if necessary, the coding was expanded by the research team. The coding was done by one of the researchers and afterwards reviewed by two others. In case of differences of opinions, the researchers met and discussed until they reached consensus. Twenty-one codes were used in total. After reaching a consensus on coding, the researchers identified three main themes and seven subthemes (Table S1 in S2 Appendix). The themes were created using inductive analysis [26]. Through continuous discussion, the authors agreed on the themes/subthemes, their interconnections, and the selection of appropriate quotations.

Data saturation in our study was achieved with 23 interviews. Nevertheless, for greater sensitivity and to prevent false saturation [27], all 30 agreed-upon interviews were conducted.

## Results

Structured interviews were conducted with 30 young doctors, including 13 who were currently undergoing a postgraduate internship and 17 who had just finished this stage of training. Most of the respondents had already chosen a specialty (63.3%) but only one of them had made that decision before the fourth year of study. Usually, the decision was reached during the postgraduate internship. The results from the interviews were analysed in three main themes: 1) factors influencing the choice of a medical specialty; 2) difficulties in choosing a specialty; 3) ways to make choosing a specialty easier.

### 1. Factors influencing the choice of medical specialty

#### 1.1. Financial factors

When indicating the factors influencing the choice of specialization, the vast majority of the respondents emphasized **earning opportunities** (and in some cases, this was the main factor), whether regarding remuneration or the prospects of earning additional money in the private sector.

*Financial factors play a crucial role*. *A calling is not enough to maintain a certain standard of life and working as a doctor*, *for some time now*, *is not so respected by society*

[F, after internship].

Not having the possibility to work in the private sector was enough to rule out a specialty for some participants.

#### 1.2. Work-related factors

The vast majority of our respondents indicated numerous influential factors related to **working conditions**, including work-life balance, heavy workload, difficult shifts, and differences in work organisation between hospital and outpatient settings. Being able to maintain **work-life balance** both during the residency training and afterwards was crucial for almost all respondents.

*Work-life balance is crucial to me*, *and having the ability to pursue other interests alongside my medical practice is important*. *I would like the flexibility to work nights if I choose to but also the option to avoid it*

[M, intern].

*I decided on family medicine*. *As a family doctor*, *you have the opportunity to earn extra*, *but you can choose to limit yourself to working from 8 to 15 with no night shifts*

[M, finished].

*I analyze how my future work will affect my personal life*. *It’s important to me that I have the time and opportunities for professional growth*, *as well as the option to work in the private sector*

[F, intern].

**Difficult night shifts, stress, and demanding patients** were also common factors actively discouraging the choice of many deficit specialties, such as internal medicine. Relatedly, some specialties are seen as being burdensome on mental or physical health.

*Specialties like surgery and orthopedics are also physically demanding—surgeries can be lengthy*, *and it’s not always possible to predict their duration*, *requiring one to stay beyond regular working hours*

[F, after internship].

Some respondents mentioned that the lack of psychological support, given the mental burden of specialties like oncology, makes them an unappealing choice.

Another frequently mentioned feature was **being able to work in both hospital and outpatient settings**. Many respondents didn’t want to be limited to working only in hospitals.

*I chose gynecology and obstetrics because it provides the possibility of establishing a private practice in the future*. *Moreover*, *this specialization involves treating both young and older patients*, *as well as both ill and healthy patients*

[F, after internship].

*In psychiatry*, *the shifts are not as demanding*, *and you can attain autonomy relatively quickly*, *allowing you to work in the private sector*. *It’s a well-paid specialty*, *and the market for psychiatrists has not yet reached saturation*, *our society needs more psychiatrists*

[M, after internship].

#### 1.3. Other factors

While economic and work-related factors were indicated as most influential, our respondents also detailed many other determinants like internship experience, personal interests and preferences, and a preferred group of patients (e.g. working only with children).

*In child psychiatry*, *you can cultivate deeper relationships with patients compared to many other specialties*. *Working with children also provides more opportunities to observe improvements in their condition*

[F, after internship].

*Family medicine gives you the possibility of being up-to-date with nearly all aspects of medicine*, *offering various work opportunities*, *and allowing for growth through interactions with different patients*

[M, after internship].

Some reported they did not possess the necessary aptitude for particular work, especially when discussing surgical specialties. Besides these more common factors, some participants mentioned how their experience from studies or postgraduate internships was influential in making their decision.

*I decided on rheumatology in large part due to the atmosphere at the rheumatology ward where I had my classes*. *It is a dream place to work*

[M, intern].

Suggestions and comments made by older physicians or their behaviour during classes were never mentioned as the main factor but were often mentioned as influential, much more often than the issue of family or peer pressure.

Another often-mentioned factor was the number and availability of residency slots. The current recruitment process was often cited as a reason for not following one’s interests:

*In the current circumstances and thresholds for more narrow specialties*, *you have to think about what is feasible not what interests you*

[M, intern].

In some cases–when talking about specialties such as radiology or pathology—lack of contact with patients was indicated. All of the respondents who decided on anaesthesiology and intensive care mentioned that the fact that both involved manual skills (such as intubation) and also more theoretical aspects of medicine was an important factor in their decision. In a few cases, the risk of legal liability was mentioned as a factor influencing participants’ decisions. However, as one respondent said:

*Legal liability is something you think about when choosing a specialty but it is not a factor but rather something you have to be aware of*

[M, after internship].

One respondent mentioned that due to her chronic disease, she is interested in a specialty connected with that illness because: *I know what it means to live with this disease and I already know a lot about diagnosis and treatment* [F, intern].

### 2. Difficulties in choosing a specialty

When respondents were asked about difficulties in choosing a medical specialty, the most frequently mentioned limitations were: 1) a lack of practical information about the daily aspects of a particular medical specialty and 2) organisation of the recruitment process.

The lack of knowledge regarding the everyday aspects of work in a given specialty included information regarding typical workday length, salary level, or the possibility of earning extra money.

*During medical studies*, *we learn about diseases–how to diagnose and treat them*. *We do not learn about working in a given specialty*

[F, after internship].

*We only see part of a specialist*’*s or resident*’*s workday*. *We come to the hospitals in the mornings when there is full staff and it is completely different than working the night shift*

[M, intern].

Another important barrier indicated by our respondents was the organisation of the recruitment process. Many participants said that due to the limited number of residency slots established by the Ministry of Health, you cannot always predict whether there will be available slots for a given specialty. One mentioned that in the current recruitment process, applicants don’t know for a long time which hospital they will end up working in. During the admission process, you have to confirm your choice knowing only the specialty, and the country region, but not knowing the specific hospital in which you will do the residency. One of the participants emphasized that in Poland there are too many specialties to choose from:

*You currently have 77 specialties to choose from. Many of them are unrecognized abroad which adds to the difficulty*

[M, intern].

Choosing a specialty is made even more challenging by the fact that some specialties are not recognized in all other European countries. For example, diabetology, angiology, or palliative medicine, which are separate specialties in Poland, are often part of broader medical specializations in many other countries [28].

### 3. Ways to make choosing specialties easier for young physicians

Most of the respondents proposed various ways to help young physicians choose a specialty. They suggested, e.g. organizing meetings with either specialists or residents in a particular medical specialty so they could honestly describe what their daily work is like, what they earn, and what possibilities for professional development they have. Another idea was to create a database of residents from various medical facilities so that before applying potential applicants could learn about working in those places. So that they might learn more about the daily work of a given specialty, many participants proposed increasing the number of classes in which students could choose a ward to study in. Furthermore, participants supported proposed changes that would allow young doctors to choose the specialty in which they would like to do their internship. While talking about postgraduate internships, some respondents also said they would like interns to be treated more like residents than students–so they could learn how it is to work in a given ward. Some respondents also proposed that there should be obligatory classes or shadowing during the night shifts in hospitals. There were also calls to change the recruitment process, such as increasing the weight of questions from a given specialty during the admission process, for example:

*If you are applying for psychiatry your result from the psychiatry questions during the Medical State Exam should count more*

[M, intern].

As mentioned above, many respondents had suggestions regarding the organization of the recruitment procedure, and they proposed how to improve it:

*It would be better if residency recruitment looked like job recruitment*. *You would apply for residency in a given hospital and they would find the candidate best suited for them*. *Currently*, *the recruitment process favours only studying for the exam and writing research papers and gives no points to people who are interested in a given specialty–who do extra shadowing or even volunteer in a given clinic*

[M, intern].

Another proposal presented by participants was to make it easier and more socially acceptable to change your specialty: *Make it more acceptable to change your specialty; abroad it is nothing strange to change your specialty after a year–after all*, *you will be practicing it for the rest of your life*, *however*, *in Poland*, *you sometimes have to suffer consequences if you change specialty too often*[F, intern].

Some of the respondents also proposed introducing a mentorship system: *It would be worthwhile to establish a mentoring system during medical studies*. *The opportunity to have a mentor who works in a specific specialization*, *in my opinion*, *is the only sensible support in making this decision*[M, intern].

Another suggested “specialty counselling” at the university where an expert would give specialty advice, maybe even introducing some sort of psychological tests to determine the best fit. Moreover, organising meetings with doctors in particular specializations or “open days for graduates”, such as job shadowing, could be beneficial.

## Discussion

This research explored the factors influencing the choice of medical specialty and our results have confirmed that these decisions are influenced by multiple factors, both economic and non-economic. It should be mentioned that no significant differences between the two subgroups of respondents (interns and those who had completed postgraduate training) were identified.

Many respondents indicated that, when choosing a specialization, they were guided by whether they would be able to work in both the public and private sectors in the future and whether they would have the flexibility to choose where they would practice medicine (hospital vs. outpatient setting). It is a common practice in Poland for physicians to be employed in more than one healthcare facility (simultaneously in the public and private sectors). In 2021, the average number of workplaces for physicians was 1.92 [2]. The main reason that the majority of medical workers undertake additional employment is unsatisfactory remuneration (still much lower than in Western European countries). The scale of dual practice and its potential consequences touches employees, employers, and the healthcare system as a whole. Numerous healthcare managers report high competition in employing health professionals and pressure for salary increases, and this shortage of health professionals is a challenge for both public and private healthcare providers. The private sector offers opportunities for higher wages and more flexible employment conditions [29].

In Poland, the remuneration of physicians is a complex and multifaceted issue, influenced by various factors such as employment sector (public or private), type of employment (civil-code contract or employment contract), type of medical facility (outpatient clinic or hospital), or medical specialty. The minimum basic salaries for residents and specialists are regulated by legal regulations and correlate to the national average income in the previous year [30]. Hospitals can pay more than the minimum; however, the frequency of this differs depending on medical specialty, type, and location of the hospital. Due to the uneven geographical distribution and shortages of medical staff, county hospitals usually have to pay more than university clinics to doctors with the same qualifications [29]. To encourage doctors to train in priority specialisations, the Minister of Health offers higher salaries to residents choosing these specializations. However, these measures have not yielded the expected results [7].

While doing residency training, most physicians work under employment contracts, whereas many specialists work under civil code contracts and are contracted by hospitals as a separate business, allowing them to avoid regulations regarding working hours [7]. In hospitals, doctors are usually paid based on their number of work hours, with shifts usually being paid extra. In some cases (especially in surgical specialties), their earnings depend on the number of conducted surgeries, and they are paid a percentage of the payment for each procedure. Informal payments happen much less often than in similar Central and Eastern European countries, with the most common informal payments taking the form of gifts rather than cash [31, 32].

While financial and career-associated factors are crucial, they are not the only determinants. Our respondents mentioned other aspects, such as work-life balance and personal motivations. They expect flexibility in working hours and better working conditions in future work, and this is important when choosing a specialty. Many researchers have confirmed changes in the top priorities of the current young generation of physicians, for whom work-life balance and personal life are crucial [33]. This fact has also been confirmed by an international study conducted among young medical professionals from 15 countries, which showed that young physicians express a strong preference for achieving a healthy balance between work and personal life, as well as valuing workplace flexibility and collaboration [34].

Very often, the choice of medical specialization was made by our respondents during the postgraduate internship, which supports the claims of the national association of medical students [35]. Additionally, our study found that the current recruitment process for postgraduate training also plays a crucial role in shaping the decisions of young doctors, with a clash between young physicians’ expectations of self-realization and the expectations of the system [36]. According to a report published in 2023 by the Supreme Audit Office, the actions taken by the Ministry of Health to increase interest in less popular, but no less important, medical specialties (including higher remuneration to graduates choosing these specializations), haven’t yielded the expected results yet [7]. The reluctance of young doctors to choose deficit specializations results from various reasons, e.g. the lack of direct contact with patients, the lack of treatment options (in the case of pathology), or the limited possibility of running a private medical practice. This was also confirmed by the results of our study.

Our findings are country-specific but are in line with the conclusions from a systematic review concerning this issue [37], which confirmed that specialties with a shortage of health professionals may attract more young doctors by increasing their interests and improving the quality of medical education. Also, studies from other countries report that changes in the training and work environment may influence young doctors’ career preferences [38]. Knowledge about these factors is also important for health workforce management because the limited possibility to train in one’s preferred specialization is one reason given by young physicians who intend to migrate [39]. In recent years, Polish young doctors, unsatisfied with barriers in their professional development, long working hours, workload, and poor working environment (including administrative burden), have organized numerous protests demanding reforms. In 2017, following strikes by junior physicians, the government introduced additional financial incentives, known as ’patriotic vouchers’, which ensured salary increases for physicians who committed to stay in the national health system for at least two years after residency training [33]. The results of our study show that focusing only on financial benefits might be inadequate when seeking to retain young doctors. Previous research also showed that intentions to migrate are associated with age, with young residents more frequently (compared with specialists) considering practicing medicine abroad [39]. Policy-makers and healthcare managers should improve working environment and ensure better training and career development opportunities.

### Policy implications

Medical specialty preferences determine the distribution of future professionals in the country. Factors and limitations affecting the choice of medical specialty should be taken into consideration by policymakers to address the deficits of doctors in certain specialties. New evidence on doctors’ medical specialty preferences could help develop policies to increase the attractiveness of deficit specialties and improve the distribution of medical staff. This study has identified several significant results that could have implications for improving postgraduate training. Our respondents indicated concrete difficulties that they faced when selecting a specialty and proposed solutions.

It is worth noting that a substantial number of respondents highlighted the current recruitment process as an important factor influencing their decision-making. To effectively implement systemic changes in the recruitment process, it is essential to engage in prior consultations and make necessary arrangements within the medical community, especially with young doctors.

Efforts aimed at enhancing access to specializations are also significant, as the difficulties regarding access to preferred specializations are indicated by young professionals as one of the crucial drivers of emigration [33, 39].

The above-described qualitative approach should be supported by subsequent quantitative studies to further explore and help better understand the nature of the determinants affecting specialty choice. There is a need for further studies to better understand the subjects’ decision-making, either through a longitudinal study measuring differences in factors over time or through a cross-sectional analysis that juxtaposes the responses of recent graduates with those of current medical students. This could offer a comprehensive understanding of how perspectives transform during the transition from student to professional.

### Study strengths

This research presents new insights into determinants and key factors of choosing medical specialties from the perspective of young doctors. One of the strengths of our study is providing new and previously uncollected data. So far, no nationwide study in this area has been conducted; rather, studies on the choice of a specific medical specialization [11] or single-center study [12] are available. The added value of this study is new evidence: our findings confirmed that work-life balance and flexible working conditions are crucial factors determining medical specialty selection by young doctors. Current changes in the key priorities and expectations of the youngest generation, such as the strong need for work-life balance and better work arrangements, are similar around the world [34], therefore, our results may also be relevant for improving postgraduate training in other countries. Moreover, the described factors and barriers indicated by our respondents could be taken into consideration by policymakers to increase the attractiveness of deficit specialties.

### Limitations

Our study is not free of limitations. First, the data gathered from interviews conducted via the platforms of Teams or Zoom may not be as comprehensive as data obtained from in-person interviews. However, in-person interviews were deemed less advantageous due to the logistical challenges they posed in terms of participant sampling, given the wide geographical distribution of potential participants. Our approach allowed us to invite participants from across the entire country.

It’s important to note that our early recruitment strategy targeted individuals belonging to specific social media groups which may lead to some bias in answers. However, the sample group was nationwide and had a country-specific gender structure indicating a satisfactory representation of young doctors. Nevertheless, we did not intend to generalise regarding the circumstances of specialty choice, but rather to qualitatively explore the previously unknown perspectives of the graduates. Our results, then, are a starting point and are intended to inform further quantitative research into this important topic. Taking into account the current crisis of the physician workforce [40], it should be underlined that the generation of our respondents (current medical graduates), will make up the future health labour market; so in depth-analysis of their perspectives and expectations is crucial for policymakers.

## Conclusions

This research provides valuable insights into the analysis of young doctors’ expectations concerning their careers. The preferences of young doctors determine the distribution of future specialists across the country. Keeping young physicians engaged and motivated in their occupations is one of the key factors in health workforce retention. Thus, both, the factors and limitations affecting the choice of medical specialty should be taken into consideration by policymakers to address the deficits of doctors in certain specialties. Better understanding young doctors’ expectations toward their medical specialties could support the development of dedicated policies to increase the attractiveness of deficit specialties and improve the distribution of healthcare professionals.

## Supporting information

S1 Appendix Consolidated criteria for reporting qualitative studies (COREQ): 32-item checklist. (PDF)

S2 Appendix Interview guide and Table S1. Themes and subthemes defined for the thematic analysis.
(PDF)
