# Supplementary material for: Factors affecting young doctors’ choice of medical specialty—A qualitative study
Source: PLoS One. 2024 Feb 1;19(2):e0297927. doi: 10.1371/journal.pone.0297927 (PMC10833556; doi:10.1371/journal.pone.0297927)
Supplement: S1 Appendix — (PDF) [file pone.0297927.s001.pdf]

S1 Appendix. Consolidated criteria for reporting qualitative studies (COREQ): 32-item checklist

No. Item	Guide questions/description	Response
Domain 1: Research team and reflexivity		
1.Personal Characteristics	Which author/s conducted the interview?	Mateusz Kulbat, Beniamin Michalik
2. Credentials	What were the researcher's credentials? E.g. PhD, MD	Mateusz Kulbat MD,<br>Beniamin Michalik MD
3. Occupation	What was their occupation at the time of the study?	Mateusz Kulbat, Medical Doctor under residency training<br>Beniamin Michalik Medical Doctor, student at the Faculty of Health Sciences
4. Gender	Was the researcher male or female?	Male
5. Experience and training	What experience or training did the researcher have?	Before undertaking the study, they had qualitative expertise through formal education and supplementary training prior the study.
Relationship with participants		
6. Relationship established	Was a relationship established prior to study commencement?	Not applicable
7. Participant knowledge of the interviewer	What did the participants know about the researcher? e.g. personal goals, reasons for doing the research	Before conducting each interview, participants were briefed on the purpose and scope of the study, which aimed to investigate the factors affecting speciality choice by young doctors.
8. Interviewer characteristics	What characteristics were reported about the interviewer/facilitator? e.g. Bias, assumptions, reasons and interests in the research topic	Interviewers are medical doctors involved in the study and with broad knowledge and interest in the topic. All participants were informed that the study protocol was approved by the Bioethical Committee of the Jagiellonian University.
Domain 2: study design		
Theoretical framework		
9. Methodological orientation and Theor	What methodological orientation was stated to underpin the study? e.g. grounded theory, discourse analysis, ethnography, phenomenology, content analysis	In this qualitative study we used thematic analysis.
Participant selection		
10. Sampling	How were participants selected? e.g. purposive, convenience, consecutive, snowball	To maximize the credibility of the research we used random sampling from people fulfilling inclusion criteria who were willing to participate.
11. Method of approach	How were participants approached? e.g. face-to-face, telephone, mail, email	Volunteers could report using online form, afterwards they were contacted via email
12. Sample size	How many participants were in the study?	30
13. Non-participation	How many people refused to participate or dropped out? Reasons?	No one refused to participate
Setting		

14. Setting of data collection	Where was the data collected? e.g. home, clinic, workplace	Interview were conducted online using platforms such as MsTeams or Zoom
15. Presence of nonparticipants	Was anyone else present besides the participants and researchers?	During the interview only the researcher and respondent were present
16. Description of sample	What are the important characteristics of the sample? e.g. demographic data, date	Characteristics of the sample are presented in Table 1
Data collection		
17. Interview guide	Were questions, prompts, guides provided by the authors? Was it pilot tested?	Yes
18. Repeat interviews	Were repeat interviews carried out? If yes, how many?	No
19. Audio/visual recording	Did the research use audio or visual recording to collect the data?	No
20. Field notes	Were field notes made during and/or after the interview?	Field notes were made during interview
21. Duration	What was the duration of the interviews or focus group?	Between 20 and 42 minutes
22. Data saturation	Was data saturation discussed?	Yes
23. Transcripts returned	Were transcripts returned to participants for comment and/or correction?	Yes
**Domain 3: analysis and findings**		
Data analysis		
24. Number of data coders	How many data coders coded the data?	1
25. Description of the coding tree	Did authors provide a description of the coding tree?	No
26. Derivation of themes	Were themes identified in advance or derived from the data?	They were derived from the data
27. Software	What software, if applicable, was used to manage the data?	NVivo Release 1.6.1.
28. Participant checking	Did participants provide feedback on the findings?	The findings were sent to the participants for their reviews.
Reporting		
29. Quotations presented	Were participant quotations presented to illustrate the themes/findings? Was each quotation identified? e.g. participant number	Yes. In each quotation we provided information about gender and whether someone is currently doing postgraduate internship or has already finished it. Participants numbers in the article were removed.
30. Data and findings consistent	Was there consistency between the data presented and the findings?	Yes
31. Clarity of major themes	Were major themes clearly presented in the findings?	Yes
32. Clarity of minor theme	Is there a description of diverse cases or discussion of minor themes?	Yes

Developed based on: Allison Tong, Peter Sainsbury, Jonathan Craig, Consolidated criteria for reporting qualitative research (COREQ): a 32-item checklist for interviews and focus groups, International Journal for Quality in Health Care, Volume 19, Issue 6, December 2007, pp. 349–357. *https://doi.org/10.1093/intqhc/mzm042*
