# Supplementary material for: Factors affecting young doctors’ choice of medical specialty—A qualitative study
Source: PLoS One. 2024 Feb 1;19(2):e0297927. doi: 10.1371/journal.pone.0297927 (PMC10833556; doi:10.1371/journal.pone.0297927)
Supplement: S2 Appendix — Themes and subthemes defined for the thematic analysis. (PDF) [file pone.0297927.s002.pdf]

S2 Appendix.

INTERVIEW GUIDE

Introduction:

1. Introduce yourself
2. Presentation of the research goal and its scientific nature.
3. Information on the duration of the study (the participant may request a break).
4. Information that the answers are confidential and the research is anonymous. Each respondent may refuse to answer certain questions or resign from participating in the study at any time.
5. Requesting and obtaining permission to participate in the study and transcript recording.

The research questions:

1. Have you already chosen a medical specialty?

If YES:

I) When did you make this decision?

II) Why did you choose this specialty?

III) What factors supported this choice?

IV) Did anything deter you from this specialty?

V) Have you considered other specialties?

If NOT:

I) What factors are you taking into account in considering your choice?

II) Have you rejected any medical specialty? If so, why?

2. What difficulties have you faced in choosing a medical specialty?

3. How could the choice of medical specialty be facilitated?

Table. S1. Themes and subthemes defined for the thematic analysis

	Themes	Subthemes
1	**Factors influencing the choice of medical specialty**	Financial factors/remuneration
		Work conditions
		Previous experience
2	**Difficulties in choosing a specialty**	Lack of experience
		Recruitment process
3	**Ways to make choosing specialties easier for young physicians**	Learning opportunities before making choosing specialty
		Changes in recruitment process
		Miscellaneous
